# Upper limits for post-wildfire floods and distinction from debris flows

**DOI:** 10.1126/sciadv.adk5713

**Published:** 2024-02-21

**Authors:** Brian A. Ebel

**Affiliations:** U.S. Geological Survey, Water Resources Mission Area, Burlington, VT, USA.

## Abstract

Upper magnitude limits and scaling with basin size for post-wildfire floods are unknown. An envelope curve was estimated defining post-wildfire flood upper limits as a function of basin area. We show the importance of separating peak flows by floods versus debris flows. Post-wildfire flood maxima are a constant 43 m^3^ s^−1^ km^−2^ for basins from 0.01 to 23 to 34 km^2^ and then declining with added basin area according to a power law relation. Intense rainfall spatial scaling may cause the envelope curve threshold at 23 to 34 km^2^. Post-wildfire flood maxima are smaller than unburned flood maxima for similar basin area. Rainstorm comparisons indicate that post-wildfire floods are triggered by smaller precipitation depths than unburned floods. Post-wildfire exceptional floods are driven by extreme rainfall rates, in contrast to post-wildfire debris flows. Runoff rates for post-wildfire envelope floods are consistent with infiltration-excess runoff. Future increases in precipitation intensity or wildfire frequency and extent could increase post-wildfire flood upper limits.

## Teaser

Post-wildfire floods can punch above their weight.

## INTRODUCTION

Elevated peak flows, including floods and debris flows, follow the flames in many wildfire-prone regions. Post-wildfire vegetation mortality and loss of surface cover reduces interception of precipitation (*1*), allowing more rainfall to reach the land surface, and can increase snow ablation rates and earlier timing of snowmelt (*2*). Vegetation mortality and stress can also reduce transpiration, potentially leading to greater groundwater recharge and wetter soil moisture conditions that promote streamflow generation (*3*). The fire-heat impulse into the soil can reduce infiltration (*4*), porosity (*5*), and surface roughness (*6*), thus intensifying surface runoff processes relative to unburned conditions. These post-wildfire process shifts may cause increases in the frequency and magnitude of floods, relative to similar rainstorms in unburned conditions (*7*–*10*). Increases in post-wildfire flood magnitudes, expressed as a ratio relative to unburned floods for similar precipitation events at the same basin, have been shown to be 10 to 100, but can be as high as 2300 times greater in the western United States (*10*). Elevated post-wildfire flood responses have been documented at many individual watersheds; however, the upper limits of post-wildfire flood magnitude, scaling of post-wildfire flood magnitude with basin area, and comparability with the upper limits of unburned floods across broad regions remain unknown.

Upper limits to flood magnitudes for unburned conditions are commonly represented as envelope curves, which relate the observed peak flood record and basin area, *A*, in a log-log diagram (*11*). Envelope curves in unburned watersheds use functional relations that represent the maximum observed volumetric flow rate (*Q*, m^3^ s^−1^) or the basin area–normalized flow rate (unit-area discharge, *Q_u_*, m^3^ s^−1^ km^−2^) as a function of *A* at regional (*11*), national (*12*), continental (*13*), and global (*14*) scales. Delineating the maximum flood expected from different burned basin sizes can aid in determining risks to human lives and infrastructure after wildfire because post-wildfire floods often strike ungauged basins. The increased prevalence and future probability of compound hazards from overlapping extreme events (*15*) and landscape disturbances (*16*), combined with increased building and fire encroachment into the wildland-urban interface (WUI) (*17*), create an imperative to advance understanding of the upper limits of post-wildfire flood magnitudes for different basin sizes.

A complication to determining the magnitude of post-wildfire floods is that observed peak flows vary by physical behavior and relative hazard depending on the fraction of total flow composed of suspended sediment and debris relative to water. Peak flows are classified into three categories by the physical deformation and flow behavior: floods, hyperconcentrated flows, and debris flows (*18*). Floods typically have <10% sediment, hyperconcentrated flows 10 to 60% sediment, and debris flows >60% sediment by volume (*18*). The addition of substantial sediment to peak flows critically alters the magnitude, velocity, and destructive power of the flow (*19*). The drastic increase in peak flow *Q* or *Q_u_* with increasing sediment concentration is referred to as “bulking” (*20*). Flow type distinction is essential when examining peak flows from burned watersheds because of the increase in flow magnitude (*21*) and the change in physical flow behavior (*22*) when floods transition to debris flows that render post facto methods to estimate flood magnitude inaccurate and misleading for debris flows (*18*). Separating observed peak flows by flow type is therefore essential for identifying differences in flow magnitude, precipitation triggering, upper limits defined by envelope curves, and how envelope curves scale with basin size.

Our primary objective is to examine and compare unburned and post-wildfire flood envelope curves by building and analyzing a database of the largest post-wildfire peak flows and separating into floods and debris flows. The largest floods from unburned watersheds, and resulting envelope curves, are compared to post-wildfire floods to understand basin area scaling of flow magnitudes and differences in the basin size. Area-normalized rates of streamflow (i.e., *Q_u_*) and characteristics of the rainfall event with inferred causality for the peak flow provide insight into differences in triggering rainfall, changes in *Q_u_* magnitude with basin scale, and the implications for the future of post-wildfire flood upper limits.

## RESULTS

### Comparison of the largest floods from burned and unburned basins

**Fig. 1.**
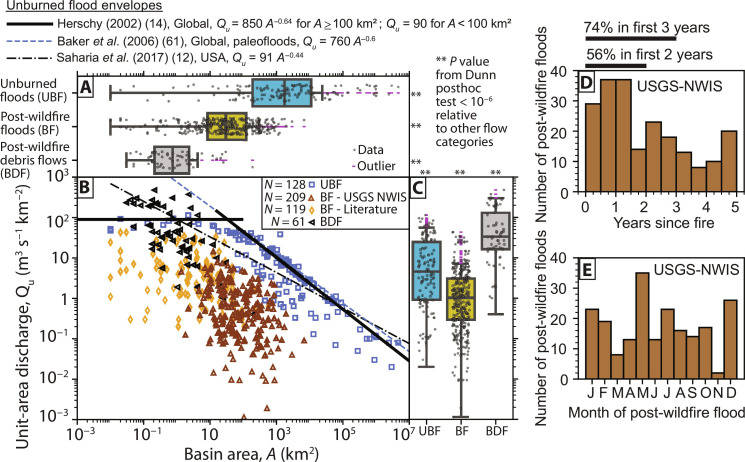
Comparison of unburned floods with post-wildfire floods and debris flows. (**A**) Box plot of basin area, *A*. (**B**) Plot of unit-area discharge (*Q_u_*) against *A* for unburned floods (UBF), post-wildfire floods (BF), and post-wildfire debris flows (BDF). *N* is the number of measurements. (**C**) Box plot of *Q_u_*. Outliers in box plots are points beyond 1.5× the interquartile range. Unburned flood envelope curves are included for comparison. The two bold, black lines mark the global unburned flood envelope curve (*14*). (**D**) Histogram of the time since fire of USGS-NWIS post-wildfire floods. (**E**) Histograms of the month of post-wildfire flood occurrence in the USGS-NWIS peak streamflow database.

The largest floods from wildfire-affected basins do not show widespread evidence of greater magnitude than unburned floods because of fire effects. Exceptional post-wildfire flood *Q_u_* are smaller than record unburned flood *Q_u_* for comparably sized basins (Dunn test, *P* = 1.6 × 10^−11^; Fig. 1, B and C) and lie well below unburned flood envelope curves (Fig. 1B). Debris flows from burned basins, in contrast, have substantially larger *Q_u_* than the largest unburned floods (Dunn test, *P* = 1.3 × 10^−7^) and post-wildfire floods (Dunn test, *P* = 3.4 × 10^−28^) (Fig. 1, B and C). The largest post-wildfire floods, examined in this study, originate from smaller basins than unburned floods (Dunn test, *P* = 1.3 × 10^−24^; Fig. 1A). Post-wildfire debris flows are triggered in smaller basins than both the largest post-wildfire floods (Dunn test, *P* = 2.1 × 10^−13^) and the largest unburned floods (Dunn test, *P* = 2.9 × 10^−41^). The largest post-wildfire floods from the U.S. Geological Survey (USGS)–National Water Information System (NWIS) peak flow database are concentrated in the first 0 to 2 years (56%) or 0 to 3 years (74%) (i.e., short-term) versus only 26% in 3 to 5 years since wildfire (i.e., mid-term) of the first 5 years post-wildfire (Fig. 1D) and are relatively evenly distributed throughout the year (Fig. 1E).

### Determination of the flood envelope curve for post-wildfire floods defining upper limits

**Fig. 2.**
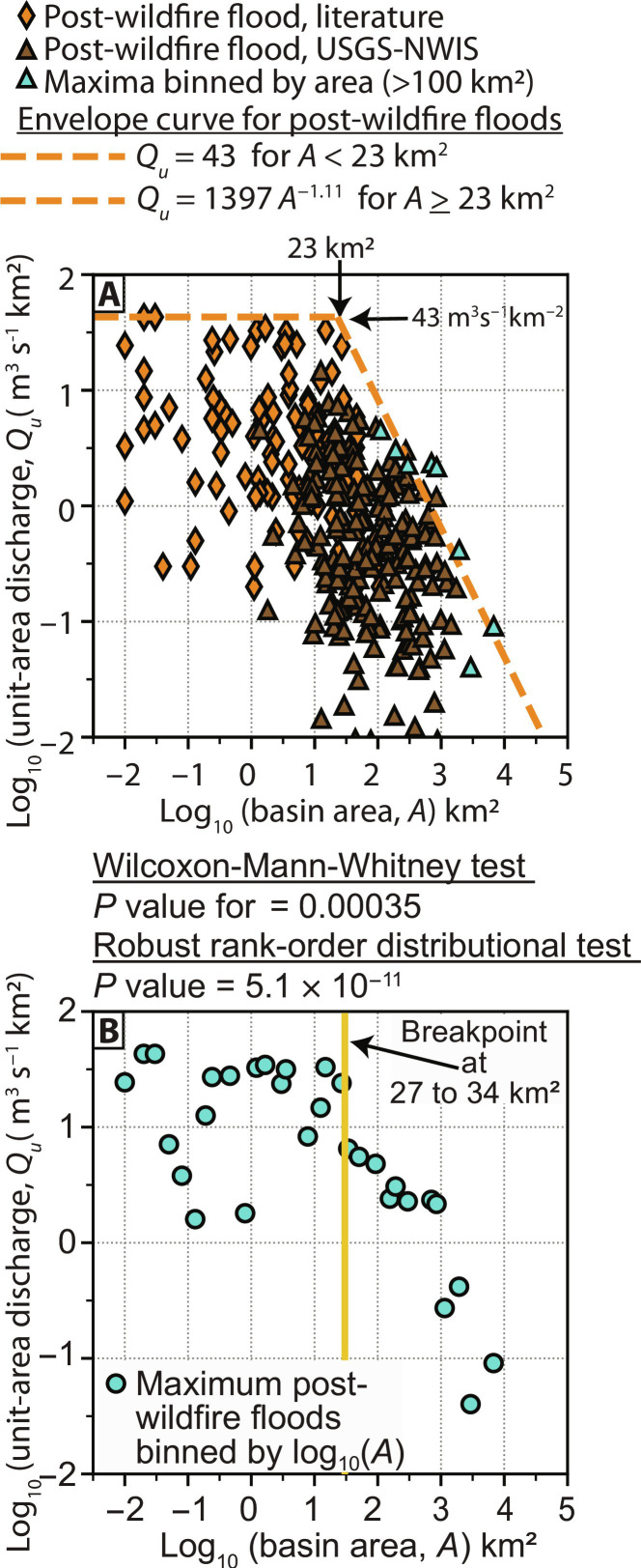
Determination of the post-wildfire flood envelope curve and independent area threshold determination. (**A**) Plot of log_10_ of unit-area discharge (*Q_u_*) against log_10_ of basin area (*A*) and the fitted post-wildfire flood envelope curve for areas with *A* > 40% burned for floods within 5 years of the wildfire. (**B**) Independent estimate of the breakpoint in log_10_(*Q_u_*) scaling with log_10_(*A*) using the Lanzante single change-point detection procedure.

Scaling of post-wildfire flood magnitudes with increasing *A* shows evidence for a basin-size threshold in scaling behavior, consistent with global (*14*) unburned envelope curves (Figs. 1B and 2, A and B). Post-wildfire flood envelope regression estimation indicates a threshold *A* of 23 km^2^ (Fig. 2A). The envelope curve of *Q_u_* is constant, regardless of *A*, for *A* from 0.01 to 23 km^2^ and then scales according to a power law for *A* ≥ 23 km^2^. An independent estimate using Lanzante single change-point detection identifies a range of the threshold *A* from 27 to 34 km^2^ (Fig. 2B). The post-wildfire envelope curve for floods shows smaller magnitude floods (i.e., *Q_u_*) at the same basin *A* relative to unburned flood envelopes. The post-wildfire flood envelope curve is defined by (Fig. 2A)

$$Q_{u}=43A<23\;{\text{km}}^{2}$$ (1A)

$$Q_{u}=1397\;A^{-1.11}A\geq 23\;{\text{km}}^{2}$$ (1B)

where *Q_u_* is unit-area discharge in m^3^ s^−1^ km^−2^ and *A* is basin area in km^2^. These analyses suggest a threshold *A* for changes in the envelope curve at 23 to 34 km^2^ for basins generating post-wildfire floods.

### Influences of rainstorm spatial scale, intensity, and total depth along with wildfire size

**Fig. 3.**
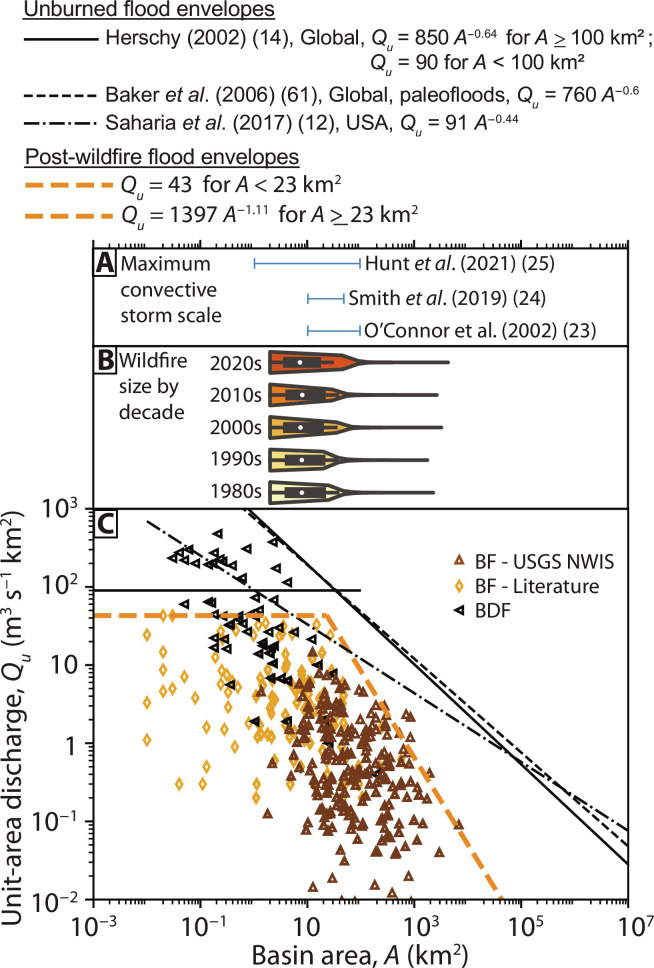
Final post-wildfire flood envelope curve compared against convective storm and wildfire area scales. (**A**) Range of convective rainstorm areal scales for individual storms. (**B**) Violin plots with embedded box plots of wildfire size in the continental United States from Monitoring Trends in Burn Severity (MTBS) (https://mtbs.gov/direct-download) separated by decade. Note that MTBS data only include fires larger than 4 km^2^ in the western United States and 2 km^2^ in the eastern United States. (**C**) Final post-wildfire flood (BF) envelope curve of unit-area discharge (*Q_u_*) against basin area (*A*) along with post-wildfire debris flows (BDF) and unburned flood envelope curves for comparison.

The largest post-wildfire flood *Q_u_* scaling with basin size may be influenced by the spatial extent of precipitation drivers and wildfire disturbance. Convective storm sizes for individual storm cells are typically 10 to 100 km^2^ (*23*–*26*), and individual storms likely only partially intersect a burned basin (Fig. 3A). Other post-wildfire flood-inducing storm types can have substantially larger spatial scales, such as atmospheric rivers and other cyclonic or frontal systems at synoptic or larger scales with maximum extents >2000 km (*27*). Wildfire size in the United States by decade from 1984 to 2021 shows that wildfire perimeter area tends to drop off between 20 and 80 km^2^ (0.75 quantile is 17 km^2^, and 0.9 quantile is 84 km^2^; Fig. 3B), although there are extreme wildfire sizes exceeding 10^3^ km^2^. The threshold *A* for scaling of the upper limits of post-wildfire floods with increasing basin size of approximately 23 to 34 km^2^ (Fig. 3C) does not clearly correspond to scaling limits for convective storm size (Fig. 3A) or wildfire perimeter area (Fig. 3B). If wildfire size was the main control on post-wildfire flood scaling thresholds for *A*, a strong correlation between the percentage of the basin burned and *Q_u_* might be expected. The correlation between the percentage of the basin burned and *Q_u_* is poor (Spearman rank correlation coefficient = 0.19, *P* = 1.8 × 10^−3^), although the correlation neglects the important influence of rainfall intensity on *Q_u_* (*28*) and *Q_u_* maxima can be dependent upon *A* (Figs. 1B and 3C).

**Fig. 4.**
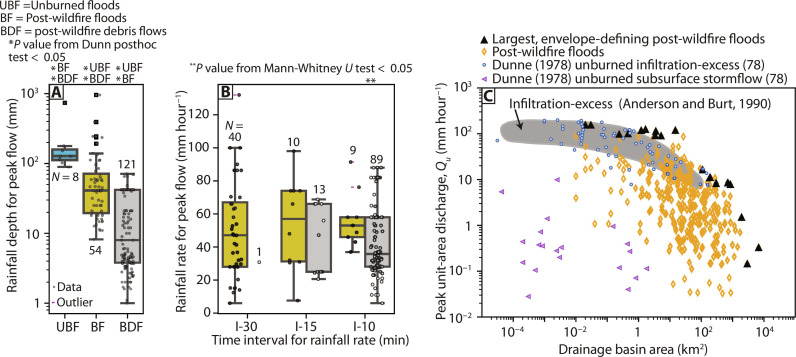
Rainfall characteristics for unburned floods, post-wildfire floods, and debris flows along with streamflow generation rates. (**A**) Box plot of rainfall depth for the storm attributed to a given peak flow. (**B**) Box plot of peak rainfall rate for the storm attributed to a given peak flow. (**C**) Rates of streamflow generation associated with post-wildfire peak unit-area discharge (*Q_u_*) compared to rates for different streamflow generation mechanisms for unburned basins (*74*, *78*) compared across basin area (*A*). *N* is the number of measurements.

**Fig. 5.**
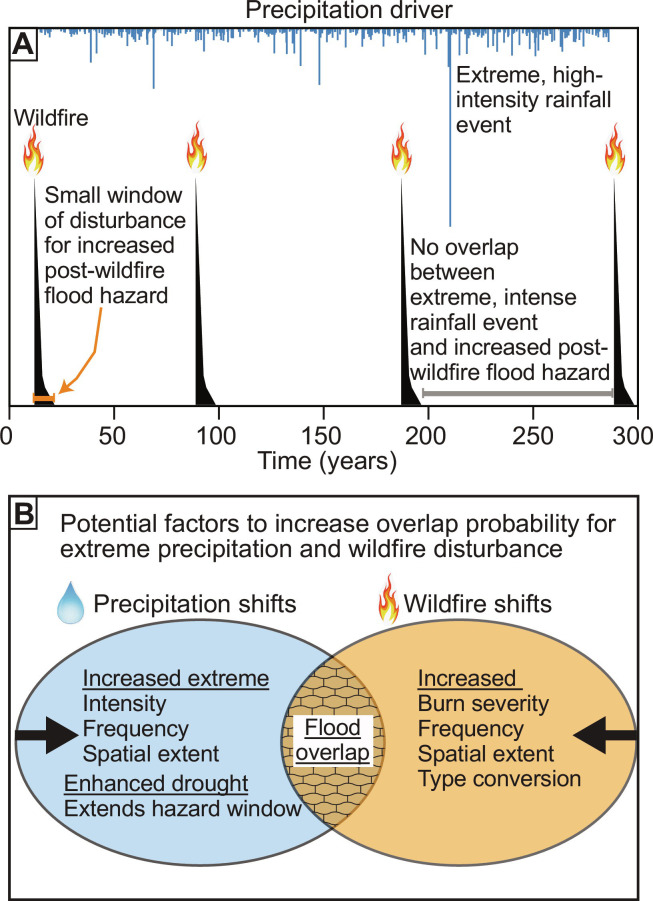
Conceptual model for primary drivers and potential future shifts for upper limits of post-wildfire floods. (**A**) Conceptual model of the interplay between the small (<5-year) window of disturbance of greatly enhanced streamflow generation and rare, extreme high-intensity rainfall events showing no overlap between elevated post-wildfire flood hazard and an extreme precipitation event driver [after (*29*, *79*)]. (**B**) Potential factors related to precipitation drivers and wildfire related to future climate shifts that could increase overlaps in space and time between the elevated post-wildfire flood window of disturbance and an extreme precipitation event driver.

Post-wildfire floods are triggered by smaller precipitation event depth than unburned floods. Comparisons of rainfall depth (Fig. 5A) indicate that the largest post-wildfire floods can be caused by smaller rainfall depths than the largest unburned floods (Dunn test, *P* = 3.7 × 10^−2^). Post-wildfire debris flows have been attributed to smaller rainfall depths than both post-wildfire floods (Dunn test, *P* = 1.6 × 10^−9^) and unburned floods (Dunn test, *P* = 5.1 × 10^−7^) (Fig. 4A). Rainfall rates separated by duration show disparate trends (Fig. 4B). Peak rainfall rates associated with post-wildfire floods were not greater than post-wildfire debris flows for 15-min rainfall rates (I-15; Mann-Whitney *U* test, *P* = 0.065) but were greater for 10-min rainfall rates (I-10; Mann-Whitney *U* test, *P* = 0.009) (Fig. 4B). Peak rainfall rates for unburned floods lacked sufficient data on the duration of the time interval and therefore could not be reliably compared.

The largest envelope curve–defining post-wildfire floods are the result of infiltration-excess runoff. This conclusion is based on comparison of post-wildfire flood *Q_u_*, expressed in mm hour^−1^, with rates of streamflow generation across drainage basin area compiled from unburned sites and grouped into streamflow generation mechanisms (Fig. 4C). Some post-wildfire floods that are not the largest envelope curve–defining floods have rates of streamflow generation more similar to saturation-excess and subsurface stormflow. This may indicate either the potential for subsurface flow processes to be important for post-wildfire flood generation or that mixtures of streamflow generation processes are operating across partially burned basins. This is reinforced by the relatively even distribution of post-wildfire floods throughout the year (Fig. 1E), although the mixing of multiple flood regimes and flood agents also influences the flood timing.

### A conceptual model of the overlap of wildfire disturbance and extreme rainfall events

Synthesis of the potential factors influencing the upper limits of post-wildfire floods points to a simplified conceptual model for why the largest post-wildfire floods are consistently smaller, in aggregate, than the largest unburned floods. The interplay between high-intensity rainfall, the duration of the effects of wildfire disturbance, and the rarity of overlap of these two factors may reduce the magnitude of the largest measured post-wildfire floods. The proposed conceptual model highlights the brief, typically <0 to 3 years (Fig. 1D), window of disturbance (*29*, *30*) after wildfire when the landscape is most prone to flood generation (Fig. 5A). The largest post-wildfire flood would be generated when the wildfire window of disturbance overlaps with a rare, extreme precipitation event that delivers high-intensity rainfall (Fig. 5A); however, this overlap in space and time is uncommon (Fig. 5, A and B). Factors that increase the probability of the overlap between the wildfire window of disturbance and extreme rainfall events include shifts in wildfire characteristics and precipitation processes (Fig. 5B), which are covered further in Discussion.

## DISCUSSION

Despite the potentially life- and property-threatening peak flows that often follow wildfires, the community has lacked envelope curves and basin-size scaling thresholds defining the upper limits of post-wildfire floods. This study identified post-wildfire flood upper limits that can aid rapid, worst-case post-fire infrastructure hazard assessment and flood extent estimates. These upper flood limits also facilitate flow type identification in the absence of sediment deposit examination (*19*) and can provide a critical accuracy assessment for extreme precipitation event flood scenario estimation with empirical (*28*) or physically based models (*31*–*33*) used after wildfire.

### Do post-wildfire floods punch above their weight?

In the sport of boxing, a fighter who punches intensely, despite their small size, is said to “punch above their weight.” Post-wildfire floods originate from smaller basins and are driven by much lower precipitation totals than the largest unburned floods. Despite their diminutive size, post-wildfire basins can deliver heavyweight floods (*10*, *34*)—they can punch above their weight. Post-wildfire basins can also deliver major floods with far smaller precipitation totals compared to unburned basins, a phenomenon observed locally (*34*) but shown here over broad regions.

Post-wildfire floods do not have to be the largest flood of record to be hazardous; if major floods are generated with much smaller rainfall totals at subhourly durations, then it could pose challenges for flood forecasting and emergency management. At the same time, the post-wildfire flood envelope curves have smaller *Q_u_* than unburned flood envelope curves, potentially because of the rare overlap of extreme rainfall events and the window of greatest hydrologic effects of wildfire (Fig. 5, A and B). The study design here does not directly address the question of whether post-wildfire floods are amplified, for a given rainstorm size, relative to the same basin in an unburned condition; instead, post-wildfire flood upper limits are assessed in aggregate. This study does provide evidence for using caution in assuming amplification by wildfire when using regional, national, or global unburned flood envelope curves to estimate the magnitude of post-wildfire floods in ungauged basins.

### Causative rainfall distinctions for post-wildfire floods relative to debris flows

The magnitude of the largest floods in unburned basins depends strongly on rainfall intensity (*12*, *35*, *36*). In semiarid to arid regions prone to wildfire such as much of the western United States, unburned floods of record tend to result from short-duration, high-intensity rainfall (*37*). Exceptional unburned floods frequently result from convective rainfall and orographic precipitation mechanisms (*35*) from warm season rainfall (*38*), although exceptional floods in the Pacific coast states have been observed in October, November, and December (*37*). The flood of record following wildfire in the western United States can originate from diverse flood agents spread throughout the year (Fig. 1E), and cool season storms can still produce intense rainfall rates (*39*). The rainfall triggers for record floods in wildfire-affected watersheds do not appear different from unburned watersheds in terms of requiring extreme rainfall intensity (*28*). Studies of record floods in unburned watersheds have indicated far greater sensitivity to rainfall intensity than basin morphometry or forest cover/disturbance (*36*, *38*). Understanding the climatology and mechanisms of intense rainfall rates may be critical for improving estimates of post-wildfire flood envelope curves and is consistent with infiltration-excess runoff generation as the dominant mechanism generating the largest post-wildfire floods. Post-wildfire debris flows, in contrast, do not require extreme rainfall intensities to initiate, and many post-wildfire debris flows are triggered by rainstorms with intensity-duration recurrence intervals of 2 years or less, with the triggering rainfall happening earlier in the storm than the peak rainfall rate (*8*). First-order dependence on extreme, high-intensity rainfall rates is a key difference in the triggering conditions for large-magnitude post-wildfire floods compared to post-wildfire debris flows, potentially because post-wildfire debris flow formation and magnitude depends strongly on sediment availability (*6*). Some observations from southern California do indicate, however, that post-wildfire debris flow magnitudes may be enhanced by greater rainfall rates at the time of triggering (*40*).

### Controls on post-wildfire flood magnitude and scaling with basin area

Post-wildfire flood *Q_u_* showed minimal correlation to the fraction of the basin burned, suggesting that pyrologic factors may be less important for post-wildfire flood *Q_u_* magnitude compared to the scale and intensity of peak rainfall rate. The dependence of *Q_u_* on *A* in unburned flood envelope curves in the United States is typically determined by the spatial scale of the triggering rainfall and basin aridity (*12*). Unburned flood *Q_u_* scaling for global envelope curves with *A* has a threshold change in scaling at ~100 km^2^ (*14*). Post-wildfire flood *Q_u_* scaling with *A* has a substantially smaller scaling threshold at 23 to 34 km^2^, which may result from many of the post-wildfire *Q_u_* data being in the western United States where intense rainfall can be smaller in areal extent and aridity is high. For example, the area enclosed by isohyets for intense, convective rainfall in the southwestern United States shows a scale change near 25 km^2^ (*41*, *42*), which corresponds to the threshold *A* for changes in post-wildfire flood *Q_u_* shown in this study. Differences in the spatial scale of storms generating intense rainfall may control thresholds in *Q_u_* scaling with *A* after wildfire. If wildfire becomes more frequent in regions with greater spatial extent of storms generating high-intensity rainfall, the threshold *A* in *Q_u_* relations could shift to larger *A*. Ultimately, the spatial scale of high-intensity rainfall is likely more important than estimates of storm spatial scale (Fig. 3A), but may be more challenging to quantify.

### Insight into post-wildfire flood generation mechanisms

Post-wildfire floods are often assumed to be generated by infiltration-excess runoff, when rainfall rate exceeds infiltration rate (*43*). For the largest post-wildfire floods that define the envelope curve, our analysis suggests that this assumption is correct (Fig. 4C). This is consistent with site-based investigations that found that the largest post-wildfire floods (*28*) were generated by infiltration-excess runoff from high-intensity rainfall. It is also congruent with recent regional analysis in Australia showing that the largest post-wildfire floods are generated by infiltration-excess runoff mechanisms (*9*). However, our analysis also suggests that relatively large post-wildfire floods may be generated by other streamflow generation mechanisms, such as saturation-excess overland flow and subsurface storm flow, or mixtures of streamflow generation mechanisms, especially in basins >1000 km^2^ (Fig. 4C). This finding is supported by small hillslope studies in burned basins that observed streamflow generation from the saturation-excess mechanism (*44*, *45*), regional studies in Australia that indicated post-wildfire saturation-excess runoff generation (*9*), and observed increases in shallow and deep subsurface streamflow from groundwater contributions (*46*). Modeling frameworks designed to predict the full spectrum of flood magnitudes following wildfire, rather than just the largest floods, may need to incorporate the full spectrum of streamflow generation mechanisms to predict post-wildfire floods, not just the infiltration-excess mechanism. It also suggests that further work on post-wildfire intense rainfall and flood climatology (*27*, *47*) would be beneficial to understanding flood generation mechanisms that have been shown to be complex in wildfire-prone, mountainous regions such as the western United States (*48*). The post-wildfire flood data analyzed here are primarily from the western United States, but the principles governing exceptional flood upper limits, basin area scaling, and flood generation mechanisms transfer readily to other burned regions (*9*). The post-wildfire flood envelope curves and conclusions derived from this analysis provide interim guidance for emergency managers until more post-wildfire flood data are available globally.

### Rare overlap of extreme precipitation events and the wildfire window of disturbance

The period of greatest threat of post-wildfire flood generation is brief. Studies in the western United States have shown that the time period immediately after the fire, even while the fire is still uncontained, may pose the greatest risk of floods (*49*) and debris flows (*50*). The post-wildfire flood risk declines substantially after the first 1 to 2 years after fire (Fig. 1D) (*51*–*53*), creating a minimal opportunity for the overlap of a rare extreme storm within the window of disturbance (Fig. 5A). This rare overlap may explain why the post-wildfire flood envelope curve results in smaller magnitude floods than unburned flood envelope curves. The largest unburned flood *Q_u_* of 90 m^3^ s^−1^ km^−2^ is equivalent to a rainfall rate of 324 mm hour^−1^ with minimal infiltration. Maximum observed rainfall rates in the United States are 300 to 320 mm hour^−1^ (*54*–*56*). An empirical post-wildfire flood model (*28*) forced with a rainfall of 324 mm hour^−1^ gives *Q_u_* of 75 m^3^ s^−1^ km^−2^. The largest post-wildfire *Q_u_* in the database used in this work was from a period that was the wettest in 75 years of record (*57*), highlighting that when exceptionally intense storms hit burned watersheds, the results could rival or potentially exceed the largest *Q_u_* from unburned watersheds. Paleoflood studies have proven useful to extend the flood record in unburned environments beyond the period of human measurement (*58*, *59*) and could discover post-wildfire paleofloods where the rare overlap of extreme precipitation and wildfire has occurred, as has been shown for post-wildfire debris flows (*60*), and provide critical information for further understanding of the upper limits of floods from burned watersheds.

### What does the future hold for post-wildfire flood upper limits?

The increased intersection of rare, extremely intense rainfall events and the brief wildfire window of disturbance could raise the upper limits (i.e., envelope curves) of post-wildfire floods. Precipitation changes, potentially relating to shifts in climate, could include precipitation extremes with greater frequency (*61*), intensity (*61*), or affecting larger areas (*62*). Drought can also lengthen the wildfire window of disturbance because of delayed vegetation recovery (*63*) and thus potentially enhance temporal overlap (Fig. 5B). Wildfire factors that could enhance the overlap include increases in annual burned area (*64*, *65*), fire frequency (*66*), burn severity (*67*), or vegetation shifts that could promote future fire ignition (Fig. 5B) (*68*). In particular, increased wildfire frequency in areas where larger mesoscale convective complexes [>50,000 km^2^; (*69*)] and supercell thunderstorms can produce near-world-record precipitation rates, for example, in the United States in Texas and the flanks of the Appalachians (*55*), could elevate the upper limits of post-wildfire flood magnitude. Some of the envelope-defining floods at larger *A* are in these types of precipitation climatology regions (table S2). An increased overlap of extreme precipitation and wildfire burn scars could require greater application of post-wildfire treatments to reduce runoff and erosion (*70*).

## MATERIALS AND METHODS

### Experimental design

A literature review was conducted using Google Scholar and Scopus to locate English language studies (including theses, reports, and journal articles) that had peak flow estimates after wildfire for floods, hyperconcentrated flows, and debris flows. No geographic or temporal restrictions were placed on data assimilation; the studies in the assembled database (*N* = 119 post-wildfire floods, *N* = 61 post-wildfire debris flows) are global in scope (see fig. S1) and range back to the 1930s. Insufficient data for hyperconcentrated flows were found to support statistical analysis, and those flows were excluded. Information from the original publication on the precipitation event for which the peak flow estimate was attributed to was noted when available; these rainfall data typically came from rain gauges. Only the largest magnitude volumetric discharge, normalized by basin area, at each basin was included in the analysis. Post-wildfire flood peak flow estimates where adjustments for flow bulking were made without documentation were not included (*71*). The classification of peak flow type in the original publication was assessed through an analysis using the rational method (*19*), and peak flow classification was qualitatively consistently correct (see the Supplementary Materials and fig. S2). The percentage burned for each basin was included in the database when available in the published report. No attempt was made to acquire additional details on the peak flow estimates or basin attributes from the publication authors.

Peak streamflow data (i.e., flood only) from the USGS-NWIS database were included to supplement the literature database. Site information for 28,528 sites was downloaded for peak streamflow data from the USGS-NWIS (https://nwis.waterdata.usgs.gov/usa/nwis/peak). These peak flow measurement locations cover the continental United States (CONUS). Drainage basins for peak streamflow sites were delineated for each site number using the Python package pyhhd (v.0.14.0) with the get_basins function that is part of the HyRiver software stack (*72*). Dropping invalid spatial geometries for drainage basins reduced the total number of sites to 26,911. The drainage basins were intersected with the Monitoring Trends in Burn Severity wildfire perimeters from 1984 to 2021 (https://mtbs.gov/direct-download) using the Python package Geopandas (v.0.12.2).

The threshold of fraction of basin burned and duration of wildfire effects on peak streamflow are not known; therefore, for the NWIS data, conservative thresholds of (i) peak flow date following fire date of less than 5 years and (ii) cumulative area burned by all wildfires in the basin in a single calendar year >40% of *A* were used. A 20% burned threshold for detectable basin response has been shown for total annual discharge (*73*), but the percentage burned for peak flow effects is not known and the higher 40% threshold used here for NWIS data enhances confidence that peak flows are influenced by wildfire effects. Peak flows from NWIS were excluded when flagged with codes in table S1 that indicated that (i) a daily average was used rather than an instantaneous peak value; (ii) all or part of the record was affected by water regulation, water diversion, urbanization, mining, agricultural changes, or channelization; (iii) the record was affected by debris dam or ice-jam breakup, hurricanes, tidal influences, or snowmelt; (iv) the record was affected by debris, mud, or hyperconcentrated flow; and (iv) other issues that were noted to substantially reduce measurement accuracy. Fire encroachment into the WUI means watersheds of mixed land use and land cover may generate or be affected by post-wildfire floods; therefore, no additional effort was taken to exclude WUI influences on basins.

Each calendar year with a total burned area fraction in a watershed >40% was considered as a separate 5-year window for peak flows, and then the maximum post-wildfire peak flow in each basin (*N* = 209) for those 5-year windows was used as a single peak flow data point for a given basin. Some of the USGS-NWIS gages that define the envelope curve are in states not always associated with wildfire effects on peak flows, such as Texas and Florida, and it is not clear that wildfire was the cause of specific elevated peak flows. The USGS-NWIS data were also filtered for a 10% burned threshold with a 5-year post-wildfire peak discharge window, a 20% burned threshold with a 10-year post-wildfire peak discharge window, and a 20% burned threshold and a 5-year post-wildfire peak discharge window to assess effects on envelope curve determination (see fig. S3).

Unburned flood data were compiled from previous databases (*14*, *35*, *54*) that include a global geographic scope from the literature that included *Q* or *Q_u_* and *A* for comparison to post-wildfire floods. Paleofloods from unburned environments (*58*) were also included for comparison. A total of 128 unburned floods were included for analysis. Information on the precipitation event presumed responsible was included when available for unburned floods. Unburned flood envelope curves (*12*, *14*, *58*) are plotted for comparison to post-wildfire flood envelope curves. *Q_u_* expressed in mm hour^−1^ was also compared against rates of runoff generation from unburned basins (*74*) to provide insight into storm timescale streamflow generation.

Some noteworthy limitations of the experimental design include (i) the geographic reach of the dataset is global but most of the sites are in the western United States (fig. S1) and therefore may not be exactly comparable to global flood envelope curves and lie closer to flood envelope curves from the United States (*12*); (ii) uncertainty in both unburned and post-wildfire peak flow measurements is not accounted for because it is seldom reported; (iii) peak flows are compared in aggregate by pooling groups (i.e., post-wildfire flood, unburned flood, and post-wildfire debris flow), but this study is not a pre- and post-fire comparison at the same basin; and (iv) separation of flood generation mechanisms into different precipitation event types, with associated climatologies, was not attempted; instead, all the peak flows are pooled by flow type and wildfire effect.

### Flood envelope curve and area scaling threshold determination

The post-wildfire basin flood envelope curve was fit using a piecewise approach (*14*). The flood envelope for post-wildfire peak discharges with *A* < 100 km^2^ was initially defined by a constant *Q_u_* equal to 43 m^3^ s^−1^ km^−2^, and basins with *A* > 100 km^2^ (*14*) were divided into eight equal bins of log_10_(*A*) and the maximum *Q_u_* in each bin was used to define the envelope curve. The 100-km^2^ breakpoint was shown in unburned flood envelope estimation (*14*) and provides a first estimate to use in refining the breakpoint for a post-wildfire flood envelope curve. A linear equation was then fit to the envelope curve points for *A* > 100 km^2^ using nonlinear least squares in the scipy.optimize.curve_fit package (v.1.10.1) in Python, with log_10_(*A*) as the dependent variable and log_10_(*Q_u_*) as the independent variable (*75*). This regression equation was then used to estimate the *A* for which *Q_u_* equals 43 m^3^ s^−1^ km^−2^ that is the breakpoint (i.e., *A* threshold) at which the envelope for maximum *Q_u_* becomes constant, regardless of area, for basins smaller than the threshold *A*.

The threshold *A* for the change in *Q_u_* scaling was independently assessed through the Lanzante single change-point detection procedure (*76*) in the trend package (v.1.1.5) in R using both a robust rank-order distributional test and a Wilcoxon-Mann-Whitney two-sample test. The nonparametric Lanzante test examines a shift in the central tendency of a time series. The data used for the Lanzante procedure were log_10_(*A*) and log_10_(*Q_u_*) for the combined database of the literature and NWIS data binned into equal log_10_(*A*) bins for the flood dataset (both literature and USGS-NWIS data). Both 20 bins and 30 bins were used, and the detected breakpoint was the same; 30 bins were used in the analysis. The Lanzante method only detects the first change point located moving from the smallest *A* to increasingly larger *A*, but multiple thresholds in scaling of *Q_u_* maxima relative to *A* may be present and would not be detected.

### Statistical analysis

Statistical comparisons of the null hypothesis of the same distribution for *Q_u_*, *A*, and storm rainfall depth used the Kruskal-Wallis test with a Dunn post hoc test to distinguish pairwise group differences using a Benjamini-Hochberg multiple comparison adjustment (*77*). The hypotheses that storm rainfall rates were greater for post-wildfire floods compared to post-wildfire debris flows and that post-wildfire debris flow bulking ratios are greater than post-wildfire flood bulking ratios were evaluated with the Mann-Whitney *U* test. The Spearman rank correlation coefficient in the scipy.stats package in Python was used as a measure of the monotonicity between log_10_(*Q_u_*) and the percentage of the basin that was burned for floods; the associated *P* value is a two-sided test with a null hypothesis that two samples have no ordinal correlation. A threshold significance level (α) of 0.05 was used for all statistical tests.

## References

[1] C. R. Stoof, R. W. Vervoort, J. Iwema, E. van den Elsen, A. J. D. Ferreira, C. J. Ritsema, Hydrological response of a small catchment burned by experimental fire. Hydrol. Earth Syst. Sci. 16, 267–285 (2012).

[2] S. K. Kampf, D. McGrath, M. G. Sears, S. R. Fassnacht, L. Kiewiet, J. C. Hammond, Increasing wildfire impacts on snowpack in the western U.S. Proc. Natl. Acad. Sci. U.S.A. 119, e2200333119 (2022). 36122238 10.1073/pnas.2200333119 PMC9522374

[3] N. M. Collar, B. A. Ebel, S. Saxe, A. J. Rust, T. S. Hogue, Implications of fire-induced evapotranspiration shifts for recharge-runoff generation and vegetation conversion in the western United States. J. Hydrol. 621, 129646 (2023).

[4] A. Cerdà, P. R. Robichaud, Fire effects on soil infiltration, in *Fire Effects on Soils and Restoration Strategies* (Science Publishers, 2009), pp. 81–103.

[5] B. A. Ebel, J. A. Moody, Parameter estimation for multiple post-wildfire hydrologic models. Hydrol. Process. 34, 4049–4066 (2020).

[6] H. Tang, L. A. McGuire, F. K. Rengers, J. W. Kean, D. M. Staley, J. B. Smith, Evolution of debris-flow initiation mechanisms and sediment sources during a sequence of postwildfire rainstorms. J. Geophys. Res. Earth 124, 1572–1595 (2019).

[7] H. W. Anderson, M. D. Hoover, K. G. Reinhart, *Forests and Water: Effects of Forest Management on Floods, Sedimentation, and Water Supply* (U.S. Forest Service, 1976).

[8] D. M. Staley, J. W. Kean, F. K. Rengers, The recurrence interval of post-fire debris-flow generating rainfall in the southwestern United States. Geomorphology 370, 107392 (2020).

[9] Z. Xu, Y. Zhang, G. Blöschl, S. Piao, Mega forest fires intensify flood magnitudes in southeast Australia. Geophys. Res. Lett. 50, e2023GL103812 (2023).

[10] D. G. Neary, G. J. Gottfried, P. F. Ffolliott, Post-wildfire watershed flood responses, paper presented at the 2nd International Wildland Fire Ecology and Fire Management Congress and 5th Symposium on Fire and Forest Meteorology, Orlando, FL, 16–20 November 2003.

[11] J. R. Crippen, C. D. Bue, *Maximum Floodflows in the Conterminous United States*. (U.S. Geological Survey, 1977).

[12] M. Saharia, P. E. Kirstetter, H. Vergara, J. J. Gourley, Y. Hong, M. Giroud, Mapping flash flood severity in the United States. J. Hydrometeorol. 18, 397–411 (2017).

[13] E. Gaume, V. Bain, P. Bernardara, O. Newinger, M. Barbuc, A. Bateman, L. Blaškovičová, G. Blöschl, M. Borga, A. Dumitrescu, I. Daliakopoulos, J. Garcia, A. Irimescu, S. Kohnova, A. Koutroulis, L. Marchi, S. Matreata, V. Medina, E. Preciso, D. Sempere-Torres, G. Stancalie, J. Szolgay, I. Tsanis, D. Velasco, A. Viglione, A compilation of data on European flash floods. J. Hydrol. 367, 70–78 (2009).

[14] R. W. Herschy, The world’s maximum observed floods. Flow Meas. Instrum. 13, 231–235 (2002).

[15] A. AghaKouchak, F. Chiang, L. S. Huning, C. A. Love, I. Mallakpour, O. Mazdiyasni, H. Moftakhari, S. M. Papalexiou, E. Ragno, M. Sadegh, Climate extremes and compound hazards in a warming world. Annu. Rev. Earth Planet. Sci. 48, 519–548 (2020).

[16] B. B. Mirus, B. A. Ebel, C. H. Mohr, N. Zegre, Disturbance hydrology: Preparing for an increasingly disturbed future. Water Resour. Res. 53, 10007–10016 (2017).

[17] V. C. Radeloff, D. P. Helmers, H. A. Kramer, M. H. Mockrin, P. M. Alexandre, A. Bar-Massada, V. Butsic, T. J. Hawbaker, S. Martinuzzi, A. D. Syphard, S. I. Stewart, Rapid growth of the US wildland-urban interface raises wildfire risk. Proc. Natl. Acad. Sci. U.S.A. 115, 3314–3319 (2018). 29531054 10.1073/pnas.1718850115 PMC5879688

[18] T. C. Pierson, *Distinguishing Between Debris Flows and Floods from Field Evidence in Small Watersheds* (U.S. Geological Survey, 2005).

[19] J. W. Kean, L. A. McGuire, F. K. Rengers, J. B. Smith, D. M. Staley, Amplification of postwildfire peak flow by debris. Geophys. Res. Lett. 43, 8545–8553 (2016).

[20] P. M. Santi, V. G. deWolfe, J. D. Higgins, S. H. Cannon, J. E. Gartner, Sources of debris flow material in burned areas. Geomorphology 96, 310–321 (2008).

[21] H. Brunkal, P. Santi, Consideration of the validity of debris-flow bulking factors. Environ. Eng. Geosci. 23, 291–298 (2017).

[22] T. C. Pierson, J. E. Costa, A rheologic classification of subaerial sediment-water flows, in *Debris Flows/Avalanches: Process, Recognition, and Mitigation*, J. E. Costa, G. F. Wieczorek, Eds. (Geological Society of America, 1987), vol. 7, pp. 1–12.

[23] J. E. O'Connor, G. E. Grant, J. E. Costa, The geology and geography of floods, in *Ancient Floods, Modern Hazards, Water Science and Application*, P. K. House, R. H. Webb, V. R. Baker, D. R. Levish, Eds. (American Geophysical Union Press, 2002), pp. 359–385.

[24] J. A. Smith, M. L. Baeck, L. Yang, J. Signell, E. Morin, D. C. Goodrich, The paroxysmal precipitation of the desert: Flash floods in the Southwestern United States. Water Resour. Res. 55, 10218–10247 (2019).

[25] A. Hunt, B. Faybishenko, B. Ghanbarian, Non-linear hydrologic organization. Nonlinear Processes Geophys. 28, 599–614 (2021).

[26] M. L. Weisman, J. B. Klemp, Characteristics of isolated convective storms, in *Mesoscale Meteorology and Forecasting*, P. S. Ray, Ed. (American Meteorological Society, 1986), pp. 331–358.

[27] N. S. Oakley, J. T. Lancaster, M. L. Kaplan, F. M. Ralph, Synoptic conditions associated with cool season post-fire debris flows in the Transverse Ranges of southern California. Nat. Hazards 88, 327–354 (2017).

[28] J. A. Moody, *An Analytical Method for Predicting Postwildfire Peak Discharges* (U.S. Geological Survey, 2011).

[29] I. P. Prosser, L. Williams, The effect of wildfire on runoff and erosion in native eucalyptus forest. Hydrol. Process. 12, 251–265 (1998).

[30] S. Saxe, T. S. Hogue, L. Hay, Characterization and evaluation of controls on post-fire streamflow response across western US watersheds. Hydrol. Earth Syst. Sci. 22, 1221–1237 (2018).

[31] R. Lew, M. Dobre, A. Srivastava, E. S. Brooks, W. J. Elliot, P. R. Robichaud, D. C. Flanagan, WEPPcloud: An online watershed-scale hydrologic modeling tool. Part I. Model description. J. Hydrol. 608, 127603 (2022).

[32] F. K. Rengers, L. A. McGuire, J. W. Kean, D. M. Staley, D. E. J. Hobley, Model simulations of flood and debris flow timing in steep catchments after wildfire. Water Resour. Res. 52, 6041–6061 (2016).

[33] F. K. Rengers, L. A. McGuire, J. W. Kean, D. M. Staley, A. M. Youberg, Progress in simplifying hydrologic model parameterization for broad applications to post-wildfire flooding and debris-flow hazards. Earth Surf. Process. Landf. 44, 3078–3092 (2019).

[34] D. J. Brogan, P. A. Nelson, L. H. MacDonald, Reconstructing extreme post-wildfire floods: A comparison of convective and mesoscale events. Earth Surf. Process. Landf. 42, 2505–2522 (2017).

[35] J. E. Costa, Hydraulics and basin morphometry of the largest flash floods in the conterminous United States. J. Hydrol. 93, 313–338 (1987).

[36] J. Pitlick, Relation between peak flows, precipitation, and physiography for five mountainous regions in the western USA. J. Hydrol. 158, 219–240 (1994).

[37] J. D. Michaud, K. K. Hirschboeck, M. Winchell, Regional variations in small-basin floods in the United States. Water Resour. Res. 37, 1405–1416 (2001).

[38] J. A. Smith, A. A. Cox, M. L. Baeck, L. Yang, P. Bates, Strange floods: The upper tail of flood peaks in the United States. Water Resour. Res. 54, 6510–6542 (2018).

[39] N. Oakley, M. Ralph, *Meteorological Conditions Associated with the Deadly 9 January 2018 Debris Flow on the Thomas Fire Burn Area Impacting Montecito, CA: A Preliminary Analysis* (Center for Western Weather and Water Extremes, Scripps Institution of Oceanography, 2018).

[40] J. W. Kean, D. M. Staley, Forecasting the frequency and magnitude of postfire debris flows across southern California. Earth’s Future 9, e2020EF001735 (2021).

[41] H. B. Osborn, W. N. Reynolds, Convective storm patterns in the southwestern United States. Hydrol. Sci. J. 8, 71–83 (1963).

[42] H. B. Osborn, K. G. Renard, J. R. Simanton, Dense networks to measure convective rainfall in the southwestern United States. Water Resour. Res. 15, 1701–1711 (1979).

[43] J. Benavides-Solorio, L. H. MacDonald, Post-fire runoff and erosion from simulated rainfall on small plots, Colorado Front Range. Hydrol. Process. 15, 2931–2952 (2001).

[44] Y. Onda, W. E. Dietrich, F. Booker, Evolution of overland flow after a severe forest fire, Point Reyes, California. Catena 72, 13–20 (2008).

[45] B. A. Ebel, J. A. Moody, D. A. Martin, Hydrologic conditions controlling runoff generation immediately after wildfire. Water Resour. Res. 48, W03529 (2012).

[46] D. M. Rey, M. A. Briggs, M. A. Walvoord, B. A. Ebel, Wildfire-induced shifts in groundwater discharge to streams identified with paired air and stream water temperature analyses. J. Hydrol. 619, 129272 (2023).

[47] M. de Orla-Barile, F. Cannon, N. S. Oakley, F. M. Ralph, A climatology of narrow cold-frontal rainbands in Southern California. Geophys. Res. Lett. 49, e2021GL095362 (2022).

[48] G. Yu, D. B. Wright, F. V. Davenport, Diverse physical processes drive upper-tail flood quantiles in the US mountain west. Geophys. Res. Lett. 49, 2022GL098855 (2022).

[49] J. A. Moody, B. A. Ebel, Hyper-dry conditions provide new insights into the cause of extreme floods after wildfire. Catena 93, 58–63 (2012).

[50] J. W. Kean, D. M. Staley, J. T. Lancaster, F. K. Rengers, B. J. Swanson, J. A. Coe, J. L. Hernandez, A. J. Sigman, K. E. Allstadt, D. N. Lindsay, Inundation, flow dynamics, and damage in the 9 January 2018 Montecito debris-flow event, California, USA: Opportunities and challenges for post-wildfire risk assessment. Geosphere 15, 1140–1163 (2019).

[51] J. A. Moody, D. A. Martin, Post-fire, rainfall intensity-peak discharge relations for three mountainous watersheds in the Western USA. Hydrol. Process. 15, 2981–2993 (2001).

[52] M. D. Kunze, J. D. Stednick, Streamflow and suspended sediment yield following the 2000 Bobcat fire, Colorado. Hydrol. Process. 20, 1661–1681 (2006).

[53] C. Wilson, S. K. Kampf, J. W. Wagenbrenner, L. H. MacDonald, Rainfall thresholds for post-fire runoff and sediment delivery from plot to watershed scales. For. Ecol. Manage. 430, 346–356 (2018).

[54] J. E. O'Connor, J. E. Costa, Spatial distribution of the largest rainfall-runoff floods from basins between 2.6 and 26,000 km^2^ in the United States and Puerto Rico. Water Resour. Res. 40, W01107 (2004).

[55] J. A. Smith, M. L. Baeck, Y. Zhange, C. A. Doswell III, Extreme rainfall and flooding from supercell thunderstorms. J. Hydrometeorol. 2, 469–489 (2001).

[56] E. M. Hansen, F. K. Schwarz, J. T. Riedel, *Probable Maximum Precipitation Estimates, Colorado River and Great Basin Drainages* (National Weather Service, 1977).

[57] K. R. Hubbert, P. M. Wohlgemuth, J. L. Beyers, M. G. Narog, R. Gerrard, Post-fire soil water repellency, hydrologic response, and sediment yield compared between grass-converted and chaparral watersheds. Fire Ecol. 8, 143–162 (2012).

[58] V. R. Baker, Palaeoflood hydrology in a global context. Catena 66, 161–168 (2006).

[59] V. R. Baker, Paleoflood hydrology: Origin, progress, prospects. Geomorphology 101, 1–13 (2008).

[60] G. A. Meyer, S. G. Wells, Fire-related sedimentation events on alluvial fans, Yellowstone National Park, USA. J. Sediment. Res. 67, 776–791 (1997).

[61] A. F. Prein, R. M. Rasmussen, K. Ikeda, C. Liu, M. P. Clark, G. J. Holland, The future intensification of hourly precipitation extremes. Nat. Clim. Change 7, 48–52 (2017).

[62] X. Tan, X. Wu, B. Liu, Global changes in the spatial extents of precipitation extremes. Environ. Res. Lett. 16, 054017 (2021).

[63] A. G. Mayor, S. Bautista, J. Llovet, J. Bellot, Post-fire hydrological and erosional responses of a Mediterranean landscpe: Seven years of catchment-scale dynamics. Catena 71, 68–75 (2007).

[64] N. J. Abram, B. J. Henley, A. Sen Gupta, T. J. Lippmann, H. Clarke, A. J. Dowdy, J. J. Sharples, R. H. Nolan, T. Zhang, M. J. Wooster, J. B. Wurtzel, K. J. Meissner, A. J. Pitman, A. M. Ukkola, B. P. Murphy, N. J. Tapper, M. M. Boer, Connections of climate change and variability to large and extreme forest fires in southeast Australia. Commun. Earth Environ. 2, 8 (2021).

[65] J. T. Abatzoglou, A. P. Williams, Impact of anthropogenic climate change on wildfire across western US forests. Proc. Natl. Acad. Sci. U.S.A. 113, 11770–11775 (2016). 27791053 10.1073/pnas.1607171113 PMC5081637

[66] A. L. Westerling, H. G. Hidalgo, D. R. Cayan, T. W. Swetnam, Warming and earlier spring increase Western U.S. forest wildfire activity. Science 313, 940–943 (2006). 16825536 10.1126/science.1128834

[67] S. A. Parks, J. T. Abatzoglou, Warmer and drier fire seasons contribute to increases in area burned at high severity in western US forests from 1985 to 2017. Geophys. Res. Lett. 47, e2020GL089858 (2020).

[68] K. Hayes, B. Buma, Effects of short-interval disturbances continue to accumulate, overwhelming variability in local resilience. Ecosphere 12, e03379 (2021).

[69] R. A. Maddox, Large-scale meteorological conditions associated with midlatitude, mesoscale convective complexes. Mon. Weather Rev. 111, 1475–1493 (1983).

[70] A. Girona-García, D. C. Vieira, J. Silva, C. Fernández, P. R. Robichaud, J. J. Keizer, Effectiveness of post-fire soil erosion mitigation treatments: A systematic review and meta-analysis. Earth Sci. Rev. 217, 103611 (2021).

[71] G. Landel, J. A. Smith, M. L. Baeck, M. Steiner, F. L. Ogden, Radar studies of heavy convective rainfall in mountainous terrain. J. Geophys. Res. Atmos. 104, 31451–31465 (1999).

[72] T. Chegini, H.-Y. Li, L. R. Leung, HyRiver: Hydroclimate data retriever. J. Open Source Soft. 6, 3175 (2021).

[73] D. W. Hallema, G. Sun, P. V. Caldwell, S. P. Norman, E. C. Cohen, Y. Liu, K. D. Bladon, S. G. McNulty, Burned forests impact water supplies. Nat. Commun. 9, 1307 (2018). 29636465 10.1038/s41467-018-03735-6 PMC5893570

[74] M. G. Anderson, T. P. Burt, Subsurface runoff, in *Process Studies in Hillslope Hydrology*, M. G. Anderson, T. P. Burt, Eds. (Wiley, 1990), pp. 365–400.

[75] A. Castellarin, Probabilistic envelope curves for design flood estimation at ungauged sites. Water Resour. Res. 43, W04406 (2007).

[76] J. R. Lanzante, Resistant, robust and non-parametric techniques for the analysis of climate data: Theory and examples, including applications to historical radiosonde station data. Int. J. Clim. 16, 1197–1226 (1996).

[77] Y. Benjamini, Y. Hochberg, Controlling the false discovery rate: A practical and powerful approach to multiple testing. J. R. Stat. Soc. B. Methodol. 57, 289–300 (1995).

[78] T. Dunne, Field studies of hillslope flow processes, in *Hillslope Hydrology*, M. J. Kirkby, Ed. (John Wiley, 1978), pp. 227–293.

[79] F. J. Swanson, Fire and geomorphic processes, in *Fire Regime and Ecosystem Properties*, H. A. Mooney, T. M. Bonnicksen, N. L. Christiansen, J. E. Lotan, W. A. Reiners, Eds. (United States Department of Agriculture, Forest Service, General Technical Report WO, United States Government Planning Office, 1981), vol. 26, pp. 401–421.

[80] B. A. Ebel, *Database of Post-Wildfire Floods and Debris Flows* (U.S. Geological Survey, 2023).

