# Supplementary material for: Upper limits for post-wildfire floods and distinction from debris flows
Source: Sci Adv. 2024 Feb 21;10(8):eadk5713. doi: 10.1126/sciadv.adk5713 (PMC10881031; doi:10.1126/sciadv.adk5713)
Supplement: Supplementary file 1 — Supplementary Text Figs. S1 to S3 Tables S1 and S2 [file sciadv.adk5713_sm.pdf]

Science Advances
AAAS

Supplementary Materials for

Upper limits for post-wildfire floods and distinction from debris flows

Brian A. Ebel

Corresponding author: Brian A. Ebel, bebel@usgs.gov

Sci. Adv. **10**, eadk5713 (2024)
DOI: 10.1126/sciadv.adk5713

This PDF file includes:

Supplementary Text
Figs. S1 to S3
Tables S1 and S2

Supplementary Text

Locations of post-wildfire flood and debris flow peak discharges

The locations of the post-wildfire floods and debris flows analyzed in this work are global in scope but originate primarily from the western United States (Fig. S1). Peak flows from countries outside the United States include locations in Spain, Australia, Israel, Canada, France, South America, South Korea, Greece, and Portugal (Fig. S1). This geographic distribution reflects current data availability and emphasizes the largest magnitude events. In the future, as wildfires affect regions across the globe and post-wildfire peak flows are measured, this database (*80*) can be updated.

Fig. S1.

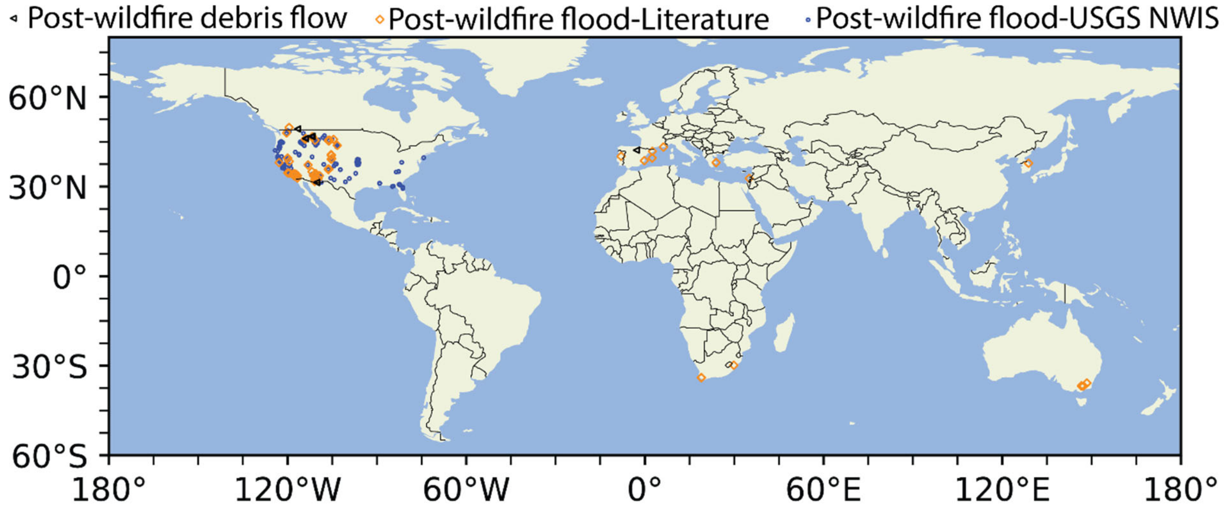

Fig. S1 Map of data points of post-wildfire debris flows, post-wildfire floods from published literature values, and post-wildfire floods from the United States Geological Survey (USGS) National Water Information System (NWIS) analyzed in this work.

Flow bulking estimation

Peak flows were separated into flow type (i.e., flood, hyperconcentrated flow, debris flow) by the original data sources. To provide an independent assessment of this flow classification, the rational equation was used to estimate flow bulking by converting the unit-area discharge Q_u into units of mm hr^{-1} and comparing to peak rainfall rates. The rational equation for bulking estimation, normalized by A, for the peak observed flow is:

$$Q_u = C\ \ I \qquad (1)$$

where Q_u is the unit-area discharge (mm hr^{-1}), I is the peak rainfall rate (mm hr^{-1}) and C is the runoff coefficient and can be considered the bulking ratio when greater than 1 (*19*) The peak flow is often attributed to the peak rainfall rate, however a peak flow (especially in the case of debris flows) can be caused by a rainfall rate preceding or succeeding the peak rainfall rate (*8*). As an approximation, runoff coefficients and bulking ratios from 0 to 1 can represent floods, from 1-2 represent hyperconcentrated flows, and >2 represent debris flows; bulking ratios for post-wildfire debris flows can be more than 50 times greater than floods (*19*). Runoff

coefficients and bulking ratios can be used as a check for the identification of flow type for measured post-wildfire peak flows, which are typically done by the morphology of flow deposits and visual markers (*18*). For bulking ratio calculations, peak flows for which there was no information on the time averaging duration of the rainfall rate or the time averaging duration of the rainfall rate was ≥6 hours were excluded from analysis.

The rational method has several key assumptions that can complicate the accuracy of the runoff coefficient estimation and become important limitations on the rational method analysis conducted in this work. Limitations of the rational method include that peak runoff happens at the time of concentration, when the entire watershed acts as the contributing area, which assumes constant and spatially uniform rainfall intensity with a time-averaging duration equivalent to the time of concentration and that the whole watershed contributes to runoff at the outlet. The watershed contributing area may be particularly problematic in basins that <100% burned or burned with a large percentage at low severity. These restrictive assumptions indicate the estimated bulking coefficients in this work should not be taken as precise estimates, but rather as a qualitative indication of whether the original post-wildfire peak flows were correctly classified.

Comparison of bulking ratios (Fig. S2A) suggests that post-wildfire flow types have been correctly characterized based on flow morphology and other indicators of flow type. This indicates that the peak flows can be analyzed as separated flood and debris flow populations based on the original flow classification from the data source. As expected, post-wildfire debris flow bulking ratios are greater than post-wildfire flood bulking ratios (Mann-Whitney U-test p_{val} 8.9e-15). Bulking ratios compared across rainfall rate averaging durations do not indicate a strong dependency of the estimate of bulking ratio on rainfall rate averaging duration for floods from burned basins (Fig. S2B). Peak rainfall rates are not systematically smaller with increasing time interval of averaging for the rainfall rate for post-wildfire floods, with the possible exception of the 60-minute rainfall rates (I-60), shown in Fig. S2C. The I-60 rainfall rates were used for the largest flood basins and the I-30 rainfall rates covered the largest range of basin sizes for post-wildfire floods (Fig. S2D), potentially because the I-30 has been considered a standard intensity for post-wildfire flood characterization and estimation (*28*).

Fig. S2.

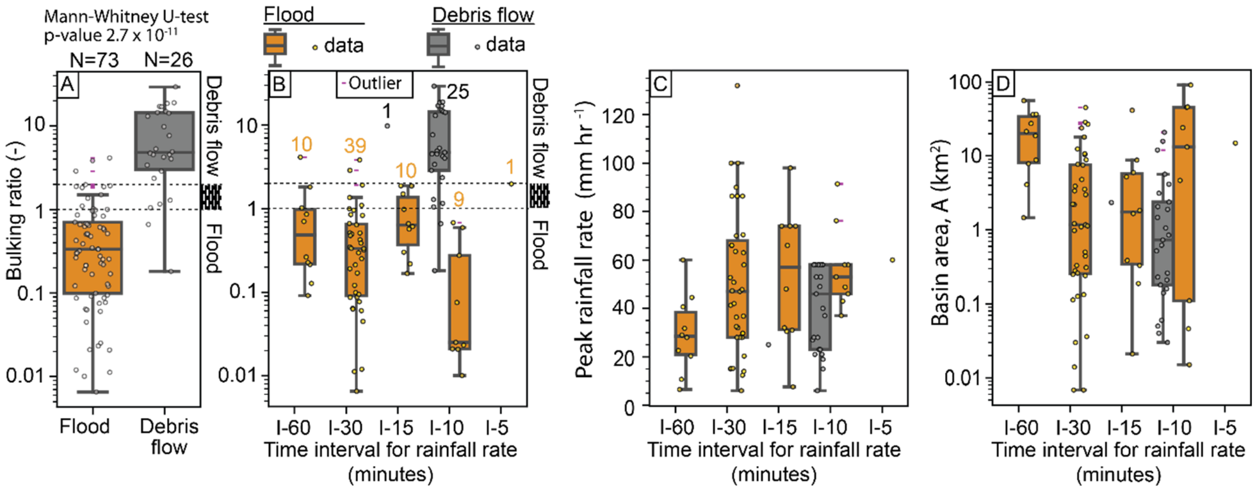

Fig. S2 Bulking of post-wildfire floods and debris flows by sediment. **(A)** Box plot of bulking ratios determined from unit-area discharge (Q_u), peak rainfall rates, and the rational equation for post-wildfire floods and debris flows. **(B)** Box plot of bulking ratio for post-wildfire floods and debris flows separated by the time average interval in minutes of the peak rainfall rate. For example, I-30 is a 30-minute rainfall rate. Outliers in box plots are points beyond 1.5 x the interquartile range. Here bulking ratios <1 indicate floods, 1-2 indicate hyperconcentrated flows (denoted by the black and white hatching), and >2 indicate debris flows; these bulking ratio mappings to flow type are approximate. N is the number of measurements. The number of measurements is shown above each box in (B) with orange text denoting floods and black text denoting debris flows. **(C)** Box plot of peak rainfall rate for post-wildfire floods and debris flows separated by the time average interval in minutes of the peak rainfall rate. **(D)** Box plot of basin area for post-wildfire floods and debris flows separated by the time average interval in minutes of the peak rainfall rate.

Sensitivity to filtering criteria for peak streamflows from the US Geological Survey National Water Information System

Different scenarios of filtering criteria used to select peak streamflows from the US Geological Survey National Water Information System (USGS-NWIS). Scenarios considered included: (1) >40% burned, <5-years post-wildfire, (2) >20% burned, <5-years post-wildfire, (3) >10% burned, <5-years post-wildfire, and (4) >10% burned, <10-years post-wildfire. Envelope-defining events from the USGS-NWIS database used 10 equal area bins for basin area, A, >100 km^2 with the exception of the >40% burned, <5-years post-wildfire sites which used 8 bins because there are so few watersheds at large scales with >40% burned.

Comparison of envelope curves for different total burned area fractions in a watershed of 10%, 20%, and 40% and the duration of the post-fire window for peak flows of 5 and 10 years showed minimal sensitivity of the estimated envelope curve to the total burned area fraction and duration of the post-fire window for peak flows between 5-10 years and 10-40% burned (Figure S3). The exponents in the power law ranged from -1.11 to -0.95 between the three percentage-burned and duration criteria of the post-wildfire window for peak flows (Figure S3).

Comparisons to threshold A using envelope curves using different criteria for post-wildfire flood data inclusion of NWIS peak flows indicated the threshold A is most likely between 20 km^2 and 40 km^2 (Fig. S3), which is congruent with the Lanzante procedure estimate.

Fig. S3.

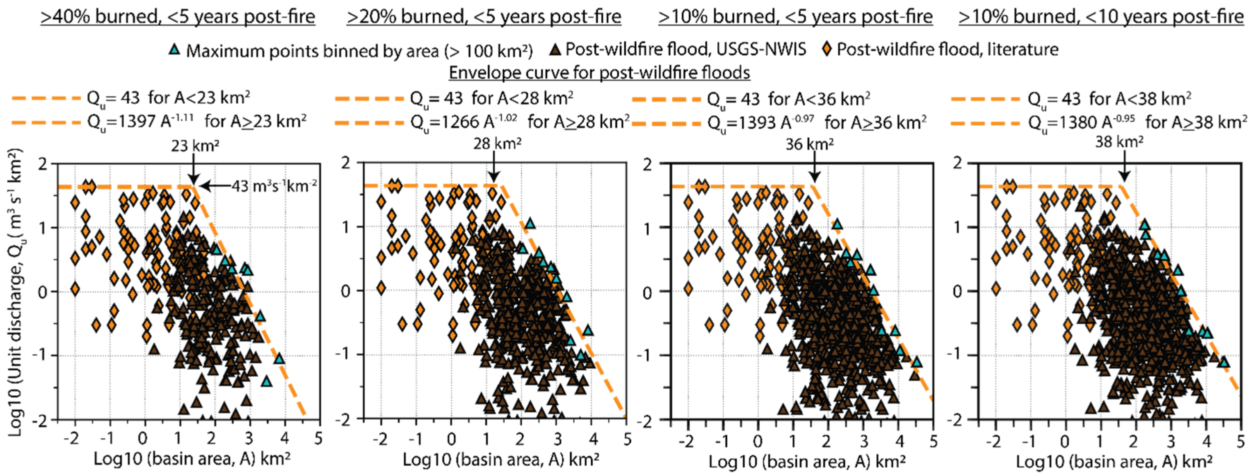

Fig. S3. Estimated post-wildfire flood envelope curves for different filtering criteria for peak streamflows from the US Geological Survey National Water Information System (USGS-NWIS). The vertical black arrow with the label denotes the intercept (basin area in km^2) of the envelope curve equation with Q_u=43 m^3 s^{-1} km^{-2}.

Table S1.

Summary statistics for basin area and unit-area discharge for peak flows in unburned and post-wildfire basins for the >40% burned, 5-years post-wildfire filtering criteria. USGS-NWIS denotes peak flows sourced from the U.S. Geological Survey National Water Information System.

		Basin area, A (km^2)			Unit-area discharge, Q_u ($m^3\ s^{-1}\ km^{-2}$)		
	Number of sites	Median	Minimum	Maximum	Median	Minimum	Maximum
Unburned floods	128	1740	0.01	4.6e6	4.5	0.02	114
Post-wildfire floods – literature	119	4.9	0.01	567	3.3	0.2	43
Post-wildfire floods-USGS NWIS	209	67.9	1.4	6782	0.45	0.001	14.7
Post-wildfire debris flows	61	0.75	0.03	190	33.3	0.4	478

Table S2.

Site information for peak streamflows defining the envelope curve from the US Geological Survey National Water Information System (USGS-NWIS). These envelope-defining events are for the >40% burned, 5-years post-wildfire filtering criteria.

USGS gage number	US state	Basin area, A (km^2)	Percentage burned (%)	Year burned
08086150	Texas	110	46	1988
09513780	Arizona	191	85	2005
11453500	California	298	68	2015
14400000	Oregon	703	75	2002
08405105	New Mexico	849	40	2011
11473900	California	1929	63	2020
02314500	Florida	2935	43	2007
06191500	Montana	6782	43	1988
